# Comparative Metabolomics and Lipidomics of Four Juvenoids Application to *Scylla paramamosain* Hepatopancreas: Implications of Lipid Metabolism During Ovarian Maturation

**DOI:** 10.3389/fendo.2022.886351

**Published:** 2022-04-27

**Authors:** Yin Fu, Fengying Zhang, Chunyan Ma, Wei Wang, Zhiqiang Liu, Wei Chen, Ming Zhao, Lingbo Ma

**Affiliations:** ^1^ Key Laboratory of East China Sea Fishery Resources Exploitation, Ministry of Agriculture and Rural Affairs, East China Sea Fisheries Research Institute, Chinese Academy of Fishery Sciences, Shanghai, China; ^2^ College of Fisheries and Life Science, Shanghai Ocean University, Shanghai, China

**Keywords:** metabolomics, lipidomics, methyl farnesoate, farnesoic acid, lipid metabolism, *Scylla paramamosain*, ovarian maturation

## Abstract

This study was the first to evaluate multiple hormonal manipulations to hepatopancreas over the ovarian development stages of the mud crab, *Scylla paramamosain*. A total of 1258 metabolites in 75 hepatopancreas explants from five female crabs were induced by juvenile hormone III (JH III), methyl farnesoate (MF), farnesoic acid (FA) and methoprene (Met), as identified from combined metabolomics and lipidomics (LC-MS/MS). 101 significant metabolites and 47 significant pathways were selected and compared for their comprehensive effects to ovarian maturation. While MF played an extensive role in lipid accumulation, JH III and Met shared similar effects, especially in the commonly and significantly elevated triglycerides and lysophospholipids (fold change≥2 and ≤0.5, VIP≥1). The significant upregulation of β-oxidation and key regulators in lipid degradation by FA (P ≤ 0.05) resulted in less lipid accumulation from this treatment, with a shift toward lipid export and energy consumption, unlike the effects of MF, JH III and Met. It was possible that MF and FA played their own unique roles and acted in synergy to modulate lipid metabolism during crab ovarian maturation. Our study yielded insights into the MF-related lipid metabolism in crustacean hepatopancreas for the overall regulation of ovarian maturation, and harbored the potential use of juvenoids to induce reproductive maturity of this economic crab species.

## 1 Introduction

The mud crab (*Scylla paramamosain*) originally distributes in the tropical Indo-Western Pacific region, and is regarded as a popular seafood in South-East Asia (1). Over the past two decades, the genus *Scylla* has gained a global interest and its farming industry has rapidly expanded over the world (2). However, under artificial cultivation conditions, one of the major challenges is that female crabs may fail to complete their normal ovarian development, causing decreased oocyte quality, less economic value and unsuccessful breeding (3). Despite the attempts modifying environmental and nutritional variables, there have been increased research efforts using hormonal manipulations in order to efficiently stimulate the ovarian development of *S. paramamosain* (4–6).

Reproduction is precisely regulated by the concentrations of sesquiterpenoids in arthropods. The sesquiterpenoid hormone methyl farnesoate (MF) is implicated in crustacean ovarian development (7). In crustaceans, MF thought to be produced by the mandibular organ (MO) serves as the key hormone in molting and reproduction regulation similar to juvenile hormone III (JH III) in insects. A positive correlation between MF levels in hemolymph and stage of reproductive maturation of the ovaries was reported in *Procambarus clarkii (* 8), freshwater crab *Oziotelphusa senex senex* (9), marine crab *Portunus trituberculatus* (10) and *S. paramamosain* (11), and estuarine crab *Neohelice granulate* (12). MF has also been reported by abundant evidence to enhance gonadal maturation and induce vitellogenesis in *Eriocheir sinesis* and other marine and fresh water crustacean species (13–16).

More than eight types of natural JHs has been found in insects, including JH 0, JH I, JH II, JH III, MF, 4-methyl JH I, bis-epoxide JH III and skipped bis-epoxide JH III (17). The most common JH is JH III, known to be produced and synthesized by corpora allata (CA) of insects (18), with pleiotropic roles in insect reproductive performance including gonadal maturation, oogenesis, vitellogenesis, reproductive behavior, pheromone and so on (19). JH signaling has also been reported to mediate fat body development and lipid storage in insect ovaries (20, 21). It is intriguing to note that MF happens to be a natural precursor and the de-epoxidized form of insect JH III, which might account for the fact that ovarian maturation in the crab *Chasmagnathus granulata* can be stimulated in the case of JH III application instead of MF, suggesting the similar effects of JH III and MF in crustaceans (22). Besides, the genes involved in JH production and degradation pathways have also been identified in water flea, shrimp and other non-insect arthropods (23).

The biosynthesis of JH and MF is conventionally divided into early mevalonate pathway and late branching steps, where farnesoic acid (FA) exists as a common precursor (24). There is also a possibility that the precursor FA itself may be another functional hormone (25). The production of FA is found upregulated to 7.5 times that of MF in MO during lobster reproduction (26). The role of FA in hepatopancreatic and/or ovarian explants in the red crab *Charybdis feriatus* (27), American lobster *Homarus americanus* (28) and penaeid shrimps *Metapenaeus ensis* (29) and *Penaeus monodon* (30) suggested that FA may be more potent than MF in stimulating vitellogenesis.

JH analogues mimicking JH usually include methoprene (Met), pyriproxyfen and hydroprene. Met is also known as isopropyl (2E, 4E)-11-methoxy-3,7,11-trimethyl-2-4-dodecadienoate, which is the most common JH analogue used as insect growth regulator to manipulate JH concentrations (31), and thus controlling developmental and reproductive processes in arthropods (32), although its efficacy proves variable and sometimes questionable (33). Nevertheless, previtellogenic Met administration is reported to significantly increase stored ovarian triglycerides and vitellogenesis in *Aedes aegypti* (34).

**Figure 1:**
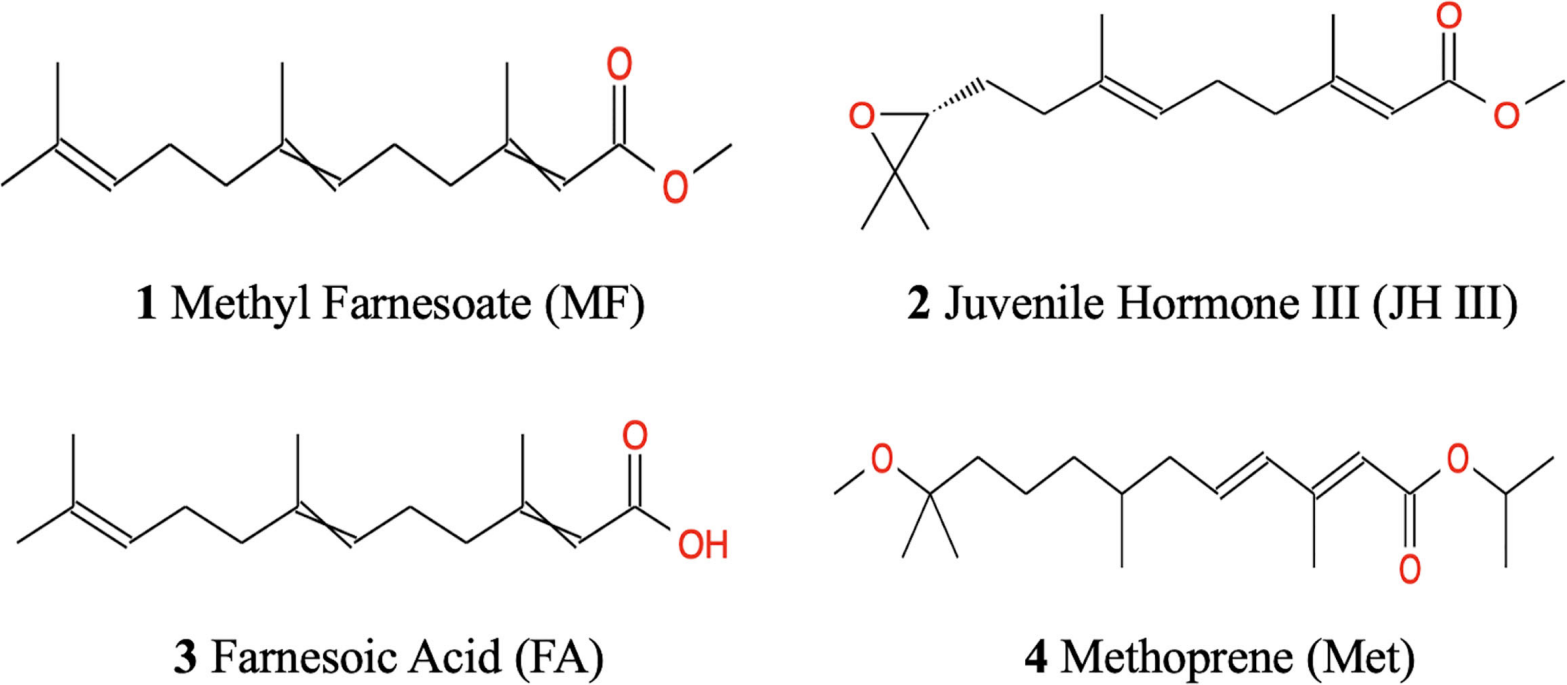
Chemical structures of MF, JH III, FA and Met.

The similar chemical structures of the above mentioned four hormonal substances are illustrated in **Figure 1**. It is believed that alterations of JH homeostasis can interfere with normal reproduction (35). Topical application of JH homologs and analogs can modulate JH homeostasis by either downregulating JH biosynthesis and transport, increasing JH signaling/catabolism, or interfering with other elements at the JH signaling pathway (36). Although JH III has not yet been identified in non-insect arthropods, JH mimics have been observed to play a role in sex development control in water flea (37). Given the similarities in endocrine control between insects and crustaceans, it is also reported that in aquatic environment, exposure to JH agonists affected estuarine crustacean species’ reproduction and larvae lipid content particularly triglycerides and sterols (38). However, there is no study yet to assess the effects of JH and its mimics to *S. paramamosain* ovarian development. Although strong evidence indicates MF and FA’s contribution to crab ovarian maturation, no current research has been done applying MF and FA directly to *S. paramamosain* to explore their metabolic roles.

Fortunately, metabolomics is able to detect intermediate metabolites, fluctuations in small molecule metabolite levels, and provides adequate data for analyses in various aspects (39). The fast-developing lipidomics is not just a single study of lipid molecules, but a comprehensive research discipline connecting metabolome and lipid accumulation. MS with extremely high resolution, sensitivity and mass precision combined with highly selective UPLC enables hundreds of lipids to be separated and identified in an unbiased way (40). It is acknowledged that crustacean hepatopancreas is the center of absorption, nutrient storage and vitellogenesis during ovarian development (41). Moreover, an abundance of enzymes in MF metabolic pathways are found in the hepatopancreas to maintain its precise titer during the ovarian development cycle of *Eriocheir sinensis* (13), *Portunus trituberculatus* (42), *Macrobrachium rosenbergii* (43), etc. Therefore, we sampled *S. paramamosain* hepatopancreas during ovarian maturation period, compared exogenous application of JH III, MF, FA and Met to hepatopancreas, and applied full spectrum metabolomics combined with lipidomics (LC-MS/MS) to systematically reveal the metabolic and lipid profiles of different treatments. This study aims to understand the MF-mediated metabolic pathways in *S. paramamosain* hepatopancreas underlying lipid accumulation and ovarian maturation, and to test *in vitro* the novel approaches of applying compounds with potential MF activity to improve reproductive maturity in this species.

## 2 Materials and Methods

### 2.1 Ethics Statement

All animal experiments in this study were conducted in accordance with the relevant national and international guidelines. Our project was approved by the East China Sea Fisheries Research Institute. In China, catching wild mud crabs from seawater does not require specific permits. Our study did not involve endangered or protected species.

### 2.2 Animals and Tissue Collection

Five healthy female *S. paramamosain* weighing 300 ± 30 g after reproductive molting were captured from the cultured population in the Ninghai Center of East China Sea Fisheries Research Institute. Once dissected, the hepatopancreas tissue samples of each crab were cut into 15 pieces, and were taken for *in vitro* experiments. Each female provided 3 similar pieces for each treatment group (random block design).

### *2.3 In Vitro* Hormone Administration

Each piece of hepatopancreas was placed in a well of a 12-well sterile culture plate (Corning Co. Ltd.^®^). Each well was previously filled with 2 mL of culture medium DMEM (Gibco) at 1g/L glucose with enhanced amino acids and vitamins, without proteins and lipids. A hormone concentration of 1 μM, just able to stimulate JH signaling pathway genes in the hepatopancreas in preliminary trials, was added. The treatments were as follows: control (n=15); MF at 1 μM (n=15); FA at 1 μM (n=15); JH III at 1 μM (n=15); Met at 1 μM (n=15). MF and FA were purchased from Echelon Biosciences, Salt Lake City, UT, USA, and JH III and Met from Sigma-Aldrich. The plates were incubated for 6 h in a shaker, under room temperature. At the end of the incubation, all the hepatopancreas pieces were suddenly frozen in liquid nitrogen before storage at -80°C until metabolomics analysis. Mixtures 1-4 were prepared as quality control (QC) to test the repeatability of analysis. The triplicate samples from the same crab hepatopancreas in each treatment group were homogenized as one sample for further analysis.

### 2.4 Sample Extraction

Hydrophilic compounds: Samples were thawed on ice. 50 mg of each sample was homogenized with 1000 μL ice-cold methanol/water (70%, v/v). Cold steel balls were used to the mix and homogenize each sample at 30 Hz for 3 min. The mixture was whirled for 1 min, and then centrifuged at 12,000 rpm, 4°C, for 10 min. The supernatant was collected for LC-MS/MS analysis.

Hydrophobic compounds: Samples were thawed on ice. 50 mg of each sample was homogenized with 1 mL mixture (including methanol, MTBE and internal standard mixture) using steel ball. The mixture for another 2 min. 500 μL water was added and whirled for 1 min, and then centrifuged at 12,000 rpm, 4°C, for 10 min. 500 μL supernatant was extracted and concentrated. The powder was dissolved with 100 μL mobile phase B, and the dissolving solution was put into the sample bottle for LC-MS/MS analysis.

### 2.5 Full Spectrum Metabolomics and Lipidomics (LC-MS/MS)

#### 2.5.1 UPLC Conditions

Both hydrophilic and hydrophobic extracts were analyzed using an LC-ESI-MS/MS system (UPLC, Shim-pack UFLC SHI- MADZU CBM A system; MS, QTRAP^®^ System). The analytical conditions were as follows. UPLC column: Waters ACQUITY UPLC HSS T3 C18 (1.8 μm, 2.1 mm*100 mm); column temperature: 40°C; flow rate: 0.4 mL/min; injection volume: 5μL; solvent system: water (0.04% acetic acid): acetonitrile (0.04% acetic acid); gradient program: 95:5 V/V at 0 min, 5:95 V/V at 11.0 min, 5:95 V/V at 12.0 min, 95:5 V/V at 12.1 min, 95:5 V/V at 14.0 min.

#### 2.5.2 ESI-Q TRAP-MS/MS

LIT and triple quadrupole (QQQ) scans were acquired on a triple quadrupole-linear ion trap mass spectrometer (QTRAP), QTRAP^®^ LC-MS/MS System, equipped with an ESI Turbo Ion-Spray interface, operating in positive and negative ion mode, and controlled by Analyst 1.6.3 software (Sciex). The ESI source operation parameters were as follows. Source temperature: 500°C; Ion spray voltage (IS): 5500 V (positive), -4500 V (negative); Ion source: gas I (GSI), gas II (GSII), curtain gas (CUR) were set at 55, 60, and 25.0 psi, respectively; The collision gas (CAD) was high. Instrument tuning and mass calibration were performed with 10 and 100 μmol/L polypropylene glycol solutions in QQQ and LIT modes, respectively. A specific set of MRM transitions was monitored for each period according to the metabolites eluted within this period.

### 2.6 Metabolomics and Lipidomics Data Analysis

Analyst 1.6.3 was used to analyze the metabolomics data. Based on the self-built target database MWDB (metware database), qualitative analysis was carried out according to the retention time of substances, ion pair information and secondary spectrum data. Metabolite quantification was completed by multiple reaction monitoring (MRM) analysis of triple quadrupole mass spectrometry. In MRM mode, the first four-stage rod first screened the precursor ions of the target substance and eliminated the precursor ions corresponding to other substances to preliminarily eliminate interference. The precursor ions were broken after collision induced ionization in the second four-stage rod, and broke according to the structural characteristics of the material to form a series of unique fragment ions. The fragment ions were filtered through the third four-stage rod to select a typical characteristic fragment ion, without interference of the untargeted ions, and the quantification was more accurate with better repeatability. After obtaining the data of different samples, MultiQuant software was used to extract ion chromatographic peaks of all metabolites, and to integrate under the peak area, and the chromatographic peaks of the same metabolite in different samples were integrated and corrected.

### 2.7 Identification of Differential Metabolites

The raw data was first unit variance scaled to prevent bias from large variance of the higher values. Simca 14.1 was used to perform Principal Component Analysis (PCA) for clustering trends, with no outliers excluded in our data. To improve group separation, Orthogonal Projections to Latent Structures Discriminant Analysis (OPLS-DA) was also applied, providing better separations between treatment groups (44). Model validity of the PCA and OPLS-DA models were checked *via* 7-fold cross-validation, which generated R2 (R2X and R2Y) and Q2 values to describe the goodness of fit and the predictive ability, respectively. Potential overfitting of the OPLS-DA model was checked by performing permutation tests. Differential metabolites were selected according to their variable importance in the projection (VIP) values from the OPLS-DA model, plus the fold change (FC) values calculated. The threshold was set at FC ≥2 and ≤0.5, and VIP≥1 to generate a list of significant discriminant metabolites between different treatments. The online database of Kyoto Encyclopedia of Genes and Genomes (KEGG; www.genome.jp/kegg/) was used for metabolite identification, biological role explanation, and pathway construction (45), and most of the affected genes and pathways are lipid metabolism-related.

### 2.8 Verification of mRNA Expression in Key Pathways

Key regulators with important roles in the production of significant different metabolites in lipid metabolism pathways were screened and the expression patterns for the encoding genes were investigated in all groups. Total RNA was was extracted from hepatopancreas samples using Total RNA Extraction Reagent (EZ-10 DNAaway RNA Extraction Kit, BBI Life Scinece), after which the quantity and quality of total RNA were assessed by NanoDrop 2000 spectrophotometer. Then, 0.2ugRNA was converted into cDNA (complementary DNA) by ReverTra Ace qPCR RT Kit (TOYOBO). Quantitative real-time polymerase chain reaction (qPCR) was performed using PerfectStart Green qPCR SuperMix (TransGen Biotech). The amplification system was performed in a 10 μL reaction volume, containing 5 μL of 2×PerfectStart Green qPCR SuperMix, 0.2 μL of forward primer (10 μM), 0.2 μL of reverse primer (10 μM), 2 μL of cDNA and 2.6 μL nuclease-free water. The reaction program for qPCR was performed as follows: initial denaturation at 50°C for 2min and then 95°C for 10 min, 40 cycles at 95°C for 5s, 60°C for 30s, followed by a continuous increase to 95°C for melting curve analysis. The gene encoding 18S rRNA was used as reference. The selected genes were those encoding hepatocyte nuclear factor 4 (HNF4), carnitine palmitoyl transferase (CPT), very-long-chain acyl-CoA dehydrogenase (VLCAD), palmitoyl protein thioesterase (PPT) and 3-ketoacyl-CoA thiolase (KAT). **Table 1** shows the primers used according to genome study (46). Three replicates were prepared for each sample. mRNA expression levels were determined using the 2^−ΔΔCT^ method (47). For each gene, the relative quantity of mRNA in arbitrary unit was interpreted as FC in comparison with the control group.

**Table 1:** Primers used for qRT-PCR.

Target mRNA	Sequence (5’-3’)
Hepatocyte nuclear factor 4-F	ATGACGAGACGAACAACGCT
Hepatocyte nuclear factor 4-R	TGCTCATCACGTTCACCTCC
Palmitoyl Protein Thioesterase-F	TCAAGTGGAGGAAGTGTGCC
Palmitoyl Protein Thioesterase-R	GTCCACCTTGTGAGAACCCC
Carnitine palmitoyl transferase-F	TGTCGCATTCCTGGGGTAAC
Carnitine palmitoyl transferase-R	TGTGCCACCACTATGTGCTT
3-ketoacyl-CoA thiolase-F	CCCCAAGCCTAAGACCACTG
3-ketoacyl-CoA thiolase-R	GATACCAGAGGCGGTTCCAG
Very-long-chain acyl-CoA dehydrogenase-F	AGATTGCGAGAGTGTGGGTG
Very-long-chain acyl-CoA dehydrogenase-R	TGCTGATGGAGGTTGCAGAG

### 2.9 Statistical Analysis

For the FCs in the raw intensities of significant metabolites and the mRNA relative expression levels of the selected genes, due to the non-parametric distribution, independent sample Kruskal Wallis tests were performed with IBM SPSS 26 to compare the differences among groups at P ≤ 0.05 as plotted in the box-and-whiskers.

## 3 Results

### 3.1 Metabolomics and Lipidomics Overview

In total, 1258 raw-data peaks were identified in hepatopancreas samples by four different hormonal treatments (FA, JH III, MF, Met) and the control group, including 474 substances from metabolomics and 784 substances from lipidomics. Representative UPLC-MS/MS total ion chromatograms (TICs) from the QC groups were presented in **Figure A1**. After integral correction, all 1258 peaks were retained (**Figure A2**). Afterwards, Z-score standarization was applied due to fairly different ranges of the original values.

### 3.2 Clustering Analysis

**Figure 2:**
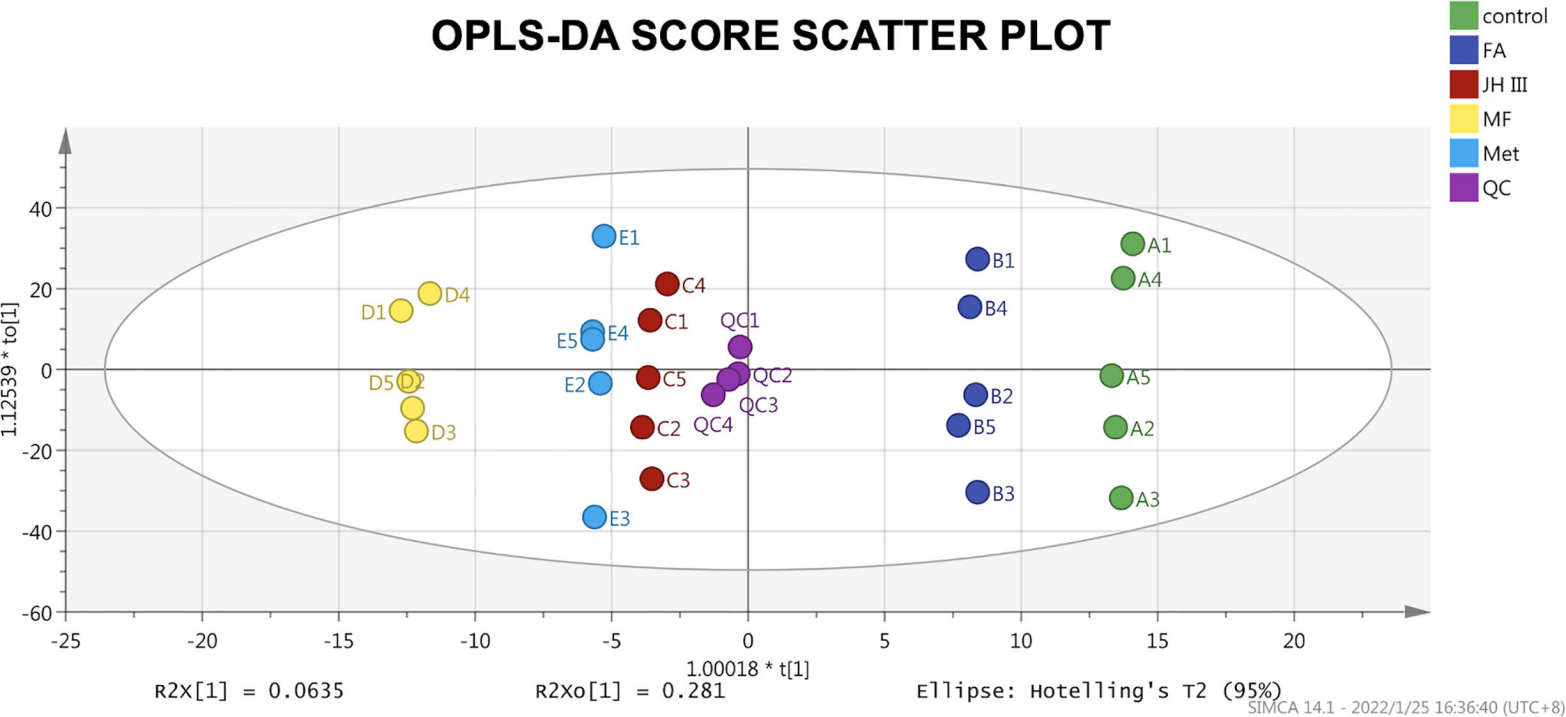
OPLS‐DA score scatter plot of all groups. Scatter color represents grouping of the same treatments as shown in the legend, and the primary ID letter A-E are corresponded with control, FA, JH III, MF and Met respectively. The score primary ID number 1-5 are corresponded with the five female crabs. Scores were all within the 95% confidence interval (Hotelling’s T2 ellipse).

The unsupervised PCA of the hepatopancreas metabolic profiles is presented in **Figure A3**. The validity of the PCA model was evaluated in terms of R2X and Q2 values, 71.3% and 54.5% respectively, with values >0.5 indicative of high accuracy. However, the different treatments were not as clearly separated as different crab samples, indicating very similar impacts of the four hormonal subjects. Another explanation is that better separation should be obtained from a larger sample size to reduce irrelevant noise signals. To further differentiate between the treatment groups, as shown by the supervised OPLS‐DA scores plot (**Figure 2**), the treatments were all clearly separated from each other on the X axis, suggesting metabolic perturbation caused by different hormonal applications. Two trends were identified from the X axis, with grouping of control and FA, as opposed to JH III, MF and Met, as separated by the Y axis. After 200 permutations for each treatment group, OPLS-DA was verified with all the intercepts of R2 on Y axis less than 0.3, and all the intercepts of Q2 on Y axis less than 0, indicating no overfitting. The three key parameters R2X, R2Y and Q2 were 0.80, 0.24 and -0.138 respectively. The R2X showed great accuracy of the model on the X axis, yet the Q2 value implied high similarity between hormonal treatments which coincides with the PCA result, indicative of their functional similarities. Both PCA and OPLS-DA results show that the QC samples are tightly gathered together showing good test quality and reliability of data, which confirms that the differences observed were more likely to result from genuine metabolite changes rather than technical errors.

### 3.3 Significant Metabolites Identification

**Figure 3:**
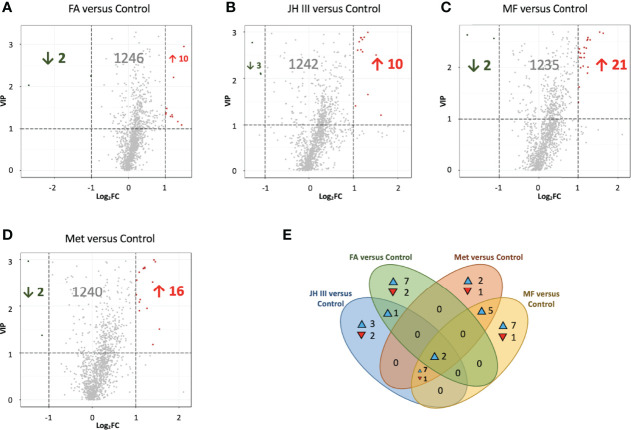
Volcano plots **(A–D)** and Venn diagram **(E)** for differential metabolites in hepatopancreas of matured *S. paramamosain*. Volcano plots were in the comparisons of **(A)** FA versus control, **(B)** JH III versus control, **(C)** MF versus control and **(D)** Met versus control. Each scatter represents a metabolite. Variable importance in projection (VIP) from OPLS-DA is used as ordinate, and Log fold change (FC) is used as abscissa. Significant metabolites were defined as FC ≥2 and ≤0.5, and VIP≥1. Significantly upregulated metabolites are in red, significantly downregulated metabolites are in green, and nonsignificantly different metabolites are in grey. Venn diagram **(E)** filtering substances differed significantly among comparison groups of FA versus control, JH III versus control, MF versus control and Met versus control. The number in the overlapping area indicates the number of common differential metabolites, whereas the number in the non-overlapping area indicates unique significant biomarkers for each treatment. ▴ and ▾ indicate the metabolites with increasing and decreasing trends respectively.

One-to-one group comparison OPLS‐DA plots are shown in **Figure A4** for differential metabolites. The number of significantly different metabolites (FC ≥2 and ≤0.5, VIP≥1) in different group comparisons is shown in **Table 2**. The differences in metabolites were further analyzed between FA and control, JH III and control, MF and control, and Met and control, in order to clearly identify the effects of each hormonal treatment. The most differential metabolites were characterized between MF and control, of which 21 were upregulated and 2 were downregulated. This is followed by Met and control, with 18 compounds differentially expressed: 16 increased and 2 decreased. JH III and control comparison reported 16 differentially metabolized biomarkers with 13 increased significantly and 3 decreased significantly. Between FA and control, 12 metabolites differed significantly, of which 10 upregulated and 2 downregulated. The differential metabolites in the four comparison groups were ranked according to their FC value (**Tables A1**–**A4**) and visualized in volcano plots according to their VIP and FC values in **Figures 3A**–**D**. Venn diagram is also presented to filter common and unique metabolites with their up/down changing trends among the four comparison groups in **Figure 3E**. There were 2 metabolites common in all four treatments when compared with control: 3-N-methyl-L-histidine and D-arabitol; 8 metabolites common in JH III, MF and Met treatments compared with control: L-homocystine, lyso-phosphatidylcholine (LPC) 18:0 and 18:3, lyso-phosphatidyl ethanolamine (LPE) 18:0, lyso-phosphatidyl glycerol (LPG) 18:1, platelet activating factor PAF C-16 (1-O-hexadecyl-2-O-acetyl-sn-glyceryl-3-phosphorylcholine), sphinganine and tryptamine. Between FA and JH III, 1 common metabolite, (±)18-HEPE (EPA-metabolite 18-hydroxyeicosapentaenoic acid), differed significantly compared with control. Between MF—control and Met—control, LPC 15:0, 16:0 and 17:0, LPE 16:0, and triglyceride (TG) (18:0/20:0/22:0) are the five common differently expressed metabolites. Of the common significant metabolites, only L-homocystine was downregulated, while the others were all upregulated. The unique differently expressed metabolites for each treatment compared with control were as follows: allysine, cysteine glutathione disulfide, LPC 16:0, 20:3, 20:4, 22:4 and O-22:4, LPE P-18:0 and phosphatidyl ethanolamine (PE) P-18:1/18:0 only for FA application; 9-hydroperoxyoctadecadienoic acid (9-HPODE), free fatty acid (FFA) (20:4), O-phospho-L-serine, oleamide and oleate only for JH III application; 18-hydroxycorticosterone, 2-(dimethylamino)guanosine, lysophosphatidic acid (LPA) 16:0, LPE (16:1(9Z)), L-phenylalanine-L-phenylalanine, TG(16:0/20:0/22:0), TG(16:0/22:0/22:0) and TG(20:2/20:1/22:0) only for MF application; L-tartaric acid, N-lactoyl-phenylalanine and tryptophyl-glutamic acid only for Met application. Of the 41 significant metabolites in Venn diagram, 31 were lipids and involved in lipid metabolic pathways. Therefore, further analysis was focused on the predominant impacts of our hormones on lipid accumulation and related mechanisms.

**Table 2:** Number of significantly differently expressed metabolites and pathways in response to different hormone treatments to the hepatopancreas of *S. paramamosain* during ovarian maturation.

Comparison	Total Sig Metabolites	Down-regulated	Up-regulated	KEGG pathways	Significant pathways
**Control — FA**	12	2	10	5	2
**Control — JH III**	16	3	13	41	10
**Control — MF**	23	2	21	27	2
**Control — Met**	18	2	16	19	1
FA — JH III	1	1	0	0	0
FA — MF	7	2	5	16	9
FA — Met	5	3	2	2	1
JH III — MF	3	1	2	3	2
JH III — Met	11	7	4	27	12
MF — Met	5	4	1	11	8

### 3.4 Lipid Accumulation and Lipid Metabolism Pathways

**Figure 4:**
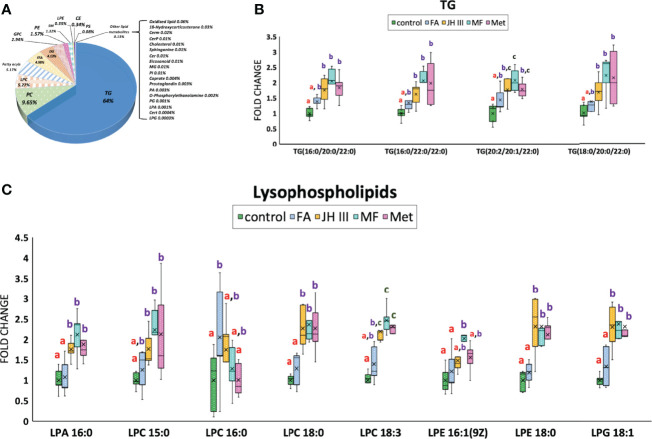
Changes of lipid accumulation and composition in hepatopancreas of *S. paramamosain* under different hormonal treatments. **(A)** Lipid subclasses identified in hepatopancreas of the mud crab and their corresponding percentages. Impact of treatments on the content of major lipid classes **(B)** TG and **(C)** lysophospholipids were presented in fold change. Different letters above boxes and whiskers denote significant difference between treatments at P ≤ 0.05 (n = 5). TG, triglyceride; PC, phosphatidyl choline; LPC, lyso-phosphatidylcholine; FFA, free fatty acid; DG, diacylglyceride; GPC, glycerylphosphorylcholine; PE, phosphatidyl ethanolamine; SM, sphingomyelin; LPE, lyso-phosphatidyl ethanolamine; CE, cholesterol ester; PS, phosphatidylserine; LPA, lyso-phosphatidic acid; LPG, lyso-phosphatidyl glycerol.

The absolute quantifications and percentages of the overall lipids in the hepatopancreas of the mud crab were categorized in a pie chart in **Figure 4A**, with more than half of the lipid accumulated being TG(64%), followed by PC (9.65%) and LPC (5.27%). Altogether four types of TGs were identified as significant metabolites and their FC values were shown in **Figure 4B**. PC was not found in significant changing metabolites. LPC and other lysophospholipids accounted for more than half of the significant lipids (16 of the 31 total significant lipids), and their FC patterns were shown in **Figure 4C**. Thus, the major changing lipids we identified were TGs and lysophospholipids.

**Figure 5:**
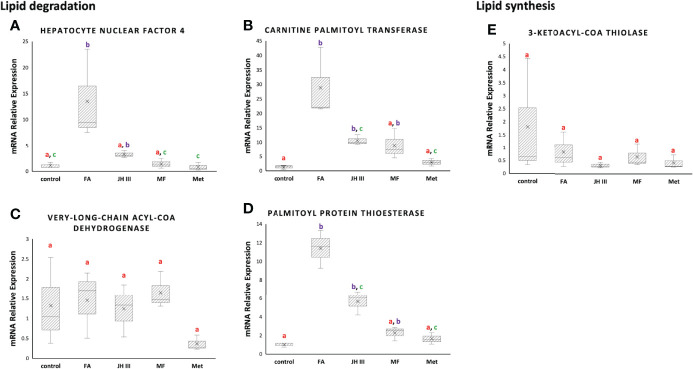
Expression of lipid metabolism genes, including the lipid degrading **(A)** hepatocyte nuclear factor 4 (*hnf4*), **(B)** carnitine palmitoyl transferase (*cpt*), **(C)** very-long-chain acyl-CoA dehydrogenase (*vlcad*) and **(D)** palmitoyl protein thioesterase (*ppt*), and the lipid synthesizing **(E)** 3-ketoacyl-CoA thiolase (*kat*) mRNA expression in hepatopancreas of *S. paramamosain* under FA, JH III, MF and Met treatments compared with control. Expression levels were calibrated for each gene using 18S rRNA as the housekeeping gene (ΔCt=Ct_gene_-Ct_18S rRNA_) and normalized using the pool of control group (ΔΔCt=ΔCt - mean ΔCt_control_). mRNA relative expression=fold change=2^-ΔΔCt^. Different letters above boxes and whiskers denote significant difference between treatments at P ≤ 0.05 (n = 3).

The relative mRNA expression of genes related to lipid metabolism in hepatopancreas of the mud crab were selected according to previous study (48) as presented in **Figure 5**, in terms of both lipid degradation **(A) (B) (C) (D)** and lipid synthesis **(E)**. The results represented the FCs of the expression of the target genes in all treatments against control. Three of the four lipid degradation genes (*hnf4*, *cpt*, *ppt*) were significantly upregulated by FA not JH III, MF or Met, opposite to the trend of TG accumulation, whereas the lipid synthesis gene (*kat*) was not stimulated by any treatment.

## 4 Discussion

### 4.1 Overview and Clustering of Metabolome and Lipidome

Several hormones have been implicated in the regulation/control of ovarian maturation in crustaceans including JH III, MF and FA (27). Our study focused on metabolomic and lipidomic changes among different juvenoids with potential MF activity, with overall 474 substances from metabolomics and 784 substances from lipidomics detected. An up-to-date study conducted using MS-based lipidomic approach in hepatopancreas for mud crab only detected a total of 390 lipid molecular species belonging to 22 lipid classes mainly glycerophospholipids, fatty acyls, glycerolipids and sphingolipids (49). Other study using untargeted metabolome analysis of hepatopancreas of the swimming crab *Portunus trituberculatus* detected a total of 628 metabolites by LC-MS (50). In hepatopancreas of *Portunus trituberculatus*, untargeted lipidomics identified a total of 1741 different lipid molecules from 32 lipid subclasses consisting of TGs, phosphatidylcholines, phosphatidylethanolamines and other lipid classes (51), which is consistent with the lipid categories we identified in **Figure 4A**. The enriched pathways in **Table 2** were typical crab hepatopancreas pathways similar to a comparative proteomics analysis of the hepatopancreas at key ovarian stages in *Eriocheir sinensis*, with protein digestion, fatty acid metabolism, prostaglandin metabolism, lipid digestion/transportation and suchlike pathways differentially expressed (52).

Among terpenoid hormones, MF might be the principal effector in *S. paramamosain* ovarian maturation in our experiment with the most metabolites expressed and pathways activated compared with the control group (**Table 2**). The OPLS‐DA clustering in **Figure 2** indicated FA had less effect than JH III, MF and Met. This is congruent with the trend from the Venn diagram in **Figure 3E**, where altogether 8 metabolites were common in JH III, MF and Met treatments compared with control apart from the 2 common significant different metabolites for all four hormonal treatments, yet there is merely 1, 0 and 0 common metabolite between FA and JH III, MF, Met respectively, when compared with control. FA showed the largest number of unique significant different metabolites upregulated, indicative of a clearly differential role of this substance as opposed to JH III, MF and Met. Structurally, **Figure 1** presented the unique carbonate acid group without esterification in FA, different from all other three sesquiterpenoids, denoting different specificities to different binding sites. Juvenile hormone acid methyltransferase (JHAMT) and farnesoic acid methyltransferase (FAMeT) are both involved in the MF biosynthesis from precursor FA in crustaceans. However, FAMeT activity in *Metapenaeus ensis* (53) and *Cancer pagurus* (54), JHAMT and FAMeT activity in *Portunus trituberculatus* (55), as requirements for the conversion of FA to MF, was solely detected in MO instead of hepatopancreas. But in *Macrobrachium rosenbergii*, FAMeT mRNA was highly expressed in the hepatopancreas (56). It is likely that FA is only produced and transformed to MF in MO not hepatopancreas in *S. paramamosain* (57). Therefore, a possible explanation is that the converting enzymes might be absent in the hepatopancreas, or inactive at the time of our hormonal addition, blocking downstream MF biosynthesis pathways from FA. This could be supported by the fact from KEGG annotation, that FA induced no other pathway apart from the only common pathway for all four hormones compared with control, pentose and glucuronate interconversions. Another explanation is the different effective concentrations. In the red crab *Charybdis feriatus*, distinct hormonal effects *in vitro* indicated the stimulatory effect of FA is more potent at low concentrations than MF and JH-III with regard to the vitellogenin gene upregulation (27). However, we only used one concentration for our experiments. Besides, MF shows inhibiting role in vitellogenesis in female crabs at middle and late vitellogenic stages, since the MF level is controlled by both anabolism and catabolism (58), whereas the role of FA reported is only for vitellogenic stimulation. The degradation pathway of MF may not be activated when there is only FA application, which also contributes to their differential effects.

The difference in metabolome and lipidome patterns following treatment with JH III, MF, FA and Met suggests that these hormones may be distinct in their effects during reproduction despite their close molecular similarity. The activity of JH III and Met should be more similar with MF activity, different from that of FA.

### 4.2 Analysis of Significant Metabolites

**Figure 3E** showed the significantly differed metabolites with the majority belonging to lipid metabolites, which mostly followed the grouping of control and FA versus JH III, MF and Met as denoted by shared letters in **Figures 4B, C**. Indeed, during the processes of reproduction, abundant lipids are accumulated and deposited in the hepatopancreas of crustaceans (59), either for energy supply or transferred to ovary to stimulate maturation, as reported in *S. paramamosain* (41). The diverse lipid categories in **Figure 4A** presented a large percentage at 64% in mud crab hepatopancreas was TG, followed by PC (9.65%) and LPC (5.27%). The significantly changed lipids we identified were also detected by Wang et al. in the hepatopancreas of the mud crab (49). Also, lipidomic analysis identified in *Daphnia magna* the changed levels of TGs, glycerophospholipids and cholesterol in females exposed to juvenoids (60). Among the significant lipid metabolites, TG and lysophospholipids were the major changing lipids. **Figure 4B** showed the four significantly altered TGs. The fatty acid compositions in TGs were the final reflection of fatty acid accumulation in crab hepatopancreas, with JH III, MF and Met significantly upregulated the types of TGs containing mostly long-chain saturated fatty acids such as 16:0, 18:0, 20:0 and 22:0 for energy storage in preparation for ovarian maturation. This is also supported by the fact that storage lipids were significantly enhanced by MF in *Daphnia magna* (61). However, FA increased TG accumulation to a much lesser extent.

LPA 16:0; LPC 15:0, 18:0, 18:3; LPE 16:1, 18:0; and LPG 18:1 also showed similar patterns under different treatments, with significant elevations in JH III, MF and Met rather than the FA and control group in **Figure 4C**, except for LPC 16:0 with a significant upregulation in FA group rather than JH III, MF and Met. Lysophospholipids are transferred from glycerophospholipids with the substitution of fatty acid chain in glycerophospholipids with hydroxyl group. Lysophospholipids are biologically active in a range of important cellular signaling pathways, functioning as ligands for G-protein-coupled receptors (62). *In vitro* and *in vivo* studies from the past decades have demonstrated or suggested the physiological functions of lysolipid signaling in reproduction, such as ovarian function, fertilization and early embryo development (63). JH III, MF and Met significantly altered membrane phospholipid metabolism, indicating common impacts leading to lysophospholipid G-protein-coupled receptor mediation and activation of related enzymes (64) with a potential activating effect to hepatopancreas. Among all significant metabolites, no juvenoid degradation products were identified. Therefore, we assume the concentration we chose was not excessive, and degradation was not significant.

### 4.3 Impacts on Lipid Metabolism Pathways

The relative mRNA expression of genes related to lipid metabolism in response to different hormonal treatments in hepatopancreas of the mud crab were presented in **Figure 5**. A significantly upregulated level of genes involved in lipid catabolism was observed in FA treatment as opposed to MF, JH III and Met when compared with control. HNF4 is the evolutionarily conserved transcription factor regulating lipid metabolism to efficiently mobilize stored fat, driving β-oxidation for energy production (65). HNF4 is a direct regulator of β-oxidation enzymes such as CPT (66), congruent with the synchronous expression patterns we found for *hnf4* and *cpt*. In **Figures 5A, B**, MF, JH III and Met showed similar patterns without significant alterations of *hnf4* and *cpt* expression compared with control, indicative of lipid reserve, whereas FA treatment significantly stimulated lipid oxidation *via hnf4* and *cpt* activation, indicative of lipid utilization. A negative association between HNF4 and TG accumulation, and even oogenesis were reported in adult *Drosophila melanogaster* (65, 67). In mosquito, JH III decreased HNF4 and lipid oxidation (48). The RNAi depletion of methoprene-tolerant receptor for JH III stimulated TG accumulation in insects (48), demonstrating the possible role of JH III in switching from lipid catabolism to lipid accumulation. MF in crustaceans is similar to JH III in insects. Therefore, the higher TG accumulation in MF, JH III and Met treatments in **Figure 4B** might result from less lipid oxidated than that in FA treatment. Finally, VLCAD is also involved in fatty acid β-oxidation, but was not affected and did not show any significant difference among all groups in **Figure 5C**.

In terms of lysophospholipids, the important intermediates in lipid metabolism and ‘lipid signal’ for various other metabolic pathways, we identified an elevated level of LPC 16:0 in FA treatment compared with other treatments and control in **Figure 4C**. This might be explained by the similarly significantly upregulated *ppt* expression level in FA group in **Figure 5D**. PPT is a soluble lipase that cleaves fatty acid chains such as palmitate, and further increases production of LPC 16:0 as catalyzed by cytosolic phospholipase A2. Free fatty acid release from storage lipids may transfer to ovary providing essential nutrients. Binding of saturated fatty acids like palmitoyl CoA (16:0) also results in activation of HNF4 (68) in concert with our data. Moreover, another important role is that PPT lies at the junction of fatty acid elongation and degradation according to KEGG, where hexadecanoyl-CoA are synthesized and then transformed by PPT into hexadecanoate for fatty acid degradation. By initiating lipid degradation during vitellogenesis and oocyte development, which was reported in the brown planthopper (69) and might also be the case of our study, the expression of *ppt* should subsequently alter the content of total lipids, explaining the differed lipid composition we found in FA group compared with MF, JH III and Met.

On the other hand, KAT is capable of catalyzing the Claisen condensation reaction between the two acyl-CoA, thereby achieving carbon chain elongation (70) and lipid synthesis (71). However, we detected no significant difference among treatments in **Figure 5E**. Therefore we assumed that the differences in TG accumulation levels were generally due to different levels of lipid degradation. Our results pointed in a direction of lipid retention reflected by the significant accumulated TGs in MF, JH III and Met treatment groups against control, contributing to effective ovarian maturation. However, for FA application, lipid reserves were utilized for energy, and might also be used in nutrient absorption or other signaling pathways. Therefore, JH III and Met shared similar activities with MF, while FA played rather different roles to MF, JH III and Met in mud crab hepatopancreas during ovarian maturation.

## 5 Conclusion

To the best of our knowledge, this is the first study investigated the impacts of FA, JH III, MF and Met to *S. paramamosain* hepatopancreas during reproduction. Altogether 75 hepatopancreas explants from five female *S. paramamosain* after reproductive molting, under 1 μM FA, JH III, MF and Met treatments and the control group, were analyzed using a combination of metabolomics and lipidomics (LC-MS/MS). MF effectively induced lipid accumulation in the mud crab hepatopancreas, especially TGs and lysophospholipids, in preparation for ovarian development. Met and JH III shared similar stimulatory roles albeit minor differences, whereas FA showed non-significant effects. The significant upregulation of β-oxidation and key regulators in lipid degradation by FA indicated a shift toward lipid export and energy consumption, as manifested by less lipid accumulated compared with MF, JH III and Met. Generally speaking, MF and FA showed their unique and irreplaceable roles in modulating lipid metabolism from the bioassays *in vivo*, but whether they act in synergy throughout the entire ovarian development cycle remained elusive. Another focal outcome was the similarity of JH III, MF and Met in nutrient accumulation, which shows implications for common features regarding the JH system between crustacean and insects, as well as the potential use of JH III, MF and Met to stimulate lipid accumulation for crab reproductive maturity. This would motivate further research to verify our findings and to clarify impacts from different stages of ovarian development and different dosages of hormones, with a larger sample size. Our study yielded important insights into the juvenoid-related metabolism controlling lipid composition in crustacean hepatopancreas, providing a linkage between hormonal and nutritional regulation during ovarian maturation, and suggesting an alternative molecular intervention to potentially induce fecundity in *S. paramamosain* with both nutritive and economic benefits to the society.

## Data Availability Statement

The raw data supporting the conclusions of this article will be made available by the authors, without undue reservation.

## Ethics Statement

The animal study was reviewed and approved by East China Sea Fisheries Research Institute.

## Author Contributions

MZ and LM designed the study; WW, WC, ZLand CM collected the animals; FZ and MZ carried out the experiment; YF performed data analysis and wrote the manuscript; MZ helped write the manuscript; LM supervised the study and provided the equipment and reagents. All authors reviewed the manuscript. All authors contributed to the article and approved the submitted version.

## Funding

Our research is supported by National Key R&D Program of China (2018YFD0901304), Program of Science and Technology Commission of Shanghai (18391900100), Special Program on Agricultural Aspect of Science and Technology Commission of Ningbo (2019B10010), Special Scientific Research Funds for Central Non-profit Institutes, Chinese Academy of Fishery Sciences (2020TD20) and China Agriculture Research System (CARS- 48).

## Conflict of Interest

The authors declare that the research was conducted in the absence of any commercial or financial relationships that could be construed as a potential conflict of interest.

## Publisher’s Note

All claims expressed in this article are solely those of the authors and do not necessarily represent those of their affiliated organizations, or those of the publisher, the editors and the reviewers. Any product that may be evaluated in this article, or claim that may be made by its manufacturer, is not guaranteed or endorsed by the publisher.
