# Supplementary material for: Comparative Metabolomics and Lipidomics of Four Juvenoids Application to Scylla paramamosain Hepatopancreas: Implications of Lipid Metabolism During Ovarian Maturation
Source: Front Endocrinol (Lausanne). 2022 Apr 27;13:886351. doi: 10.3389/fendo.2022.886351 (PMC9094423; doi:10.3389/fendo.2022.886351)
Supplement: Supplementary file 1 [file DataSheet_1.docx]

Supplementary Material

**Supplementary Table A1**. Significantly differently expressed metabolites in FA versus control

| Compounds | Class | VIP | Fold Change | Type |
| --- | --- | --- | --- | --- |
| D-Arabitol | Carbohydrate metabolomics | 2.949 | 2.821 | up |
| Cysteine glutathione disulfide | Amino Acid metabolomics | 1.091 | 2.705 | up |
| LPC(O-22:4/0:0) | LPC-O | 1.172 | 2.521 | up |
| 3-N-Methyl-L-Histidine | Amino Acid metabolomics | 2.217 | 2.324 | up |
| LPC(20:3/0:0) | LPC | 1.285 | 2.280 | up |
| LPC(22:4/0:0) | LPC | 1.330 | 2.257 | up |
| LPC(20:4/0:0) | LPC | 1.292 | 2.198 | up |
| LPC(16:0/0:0) | LPC | 1.481 | 2.047 | up |
| (±)18-HEPE | Eicosanoid | 1.378 | 2.021 | up |
| LPE(P-18:0/0:0) | LPE-P | 1.347 | 2.007 | up |
| Allysine | Organic Acid And Its Derivatives | 2.249 | 0.495 | down |
| PE(P-18:1/18:0) | PE-P | 2.030 | 0.155 | down |

**Supplementary Table A2**. Significantly differently expressed metabolites in JH III versus control

| Compounds | Class | VIP | Fold Change | Type |
| --- | --- | --- | --- | --- |
| (±)18-HEPE | Eicosanoid | 1.209 | 3.078 | up |
| D-Arabitol | Carbohydrate metabolomics | 2.502 | 2.851 | up |
| 3-N-Methyl-L-Histidine | Amino Acid metabolomics | 1.652 | 2.508 | up |
| Tryptamine | Tryptamines And Its Derivatives | 2.989 | 2.507 | up |
| sphinganine | Lipids | 2.884 | 2.370 | up |
| Lysope 18:0 | LipidsOthersPhospholipid | 2.572 | 2.310 | up |
| Lysopg 18:1 | LipidsOthersPhospholipid | 2.835 | 2.301 | up |
| Lysopc 18:0 | LipidsOthersPhospholipid | 2.876 | 2.261 | up |
| 9-Hpode | Lipids | 2.615 | 2.250 | up |
| PAF C-16 | Lipids | 2.875 | 2.244 | up |
| Lysopc 18:3 | LipidsOthersPhospholipid | 2.788 | 2.192 | up |
| O-Phospho-L-Serine | Amino Acid metabolomics | 2.606 | 2.138 | up |
| FFA(20:4) | Eicosanoid | 1.408 | 2.068 | up |
| Oleamide | Lipids_Fatty Acids | 2.092 | 0.468 | down |
| Oleate | Lipids | 2.112 | 0.466 | down |
| L-Homocystine | Amino Acid metabolomics | 2.769 | 0.409 | down |

**Supplementary Table A3**. Significantly differently expressed metabolites in MF versus control

| Compounds | Class | VIP | Fold Change | Type |
| --- | --- | --- | --- | --- |
| sphinganine | Lipids | 2.659 | 3.093 | up |
| Tryptamine | Tryptamines And Its Derivatives | 2.682 | 2.914 | up |
| Lysopc 18:3 | LipidsOthersPhospholipid | 2.531 | 2.466 | up |
| D-Arabitol | Carbohydrate metabolomics | 2.002 | 2.374 | up |
| Lysopg 18:1 | LipidsOthersPhospholipid | 2.575 | 2.374 | up |
| Lysopc 18:0 | LipidsOthersPhospholipid | 2.540 | 2.361 | up |
| Lysopc 17:0 | LipidsOthersPhospholipid | 1.887 | 2.353 | up |
| PAF C-16 | Lipids | 2.524 | 2.338 | up |
| Lysope 18:0 | LipidsOthersPhospholipid | 2.369 | 2.309 | up |
| TG(18:0/20:0/22:0) | TG | 2.189 | 2.225 | up |
| Lysopc 15:0 | LipidsOthersPhospholipid | 2.271 | 2.225 | up |
| Lysopc 16:0 | LipidsOthersPhospholipid | 2.384 | 2.224 | up |
| Lysope 16:0 | LipidsOthersPhospholipid | 2.021 | 2.187 | up |
| Lysopa 16:0 | LipidsOthersPhospholipid | 2.186 | 2.117 | up |
| 18-Hydroxycorticosterone | Lipids | 1.990 | 2.090 | up |
| TG(20:2/20:1/22:0) | TG | 2.273 | 2.080 | up |
| TG(16:0/22:0/22:0) | TG | 2.192 | 2.065 | up |
| TG(16:0/20:0/22:0) | TG | 2.356 | 2.045 | up |
| 3-N-Methyl-L-Histidine | Amino Acid metabolomics | 1.618 | 2.037 | up |
| LysoPE(16:1(9Z)-0:0) | Lipids_Fatty Acids | 2.294 | 2.018 | up |
| Phe-Phe | Amino Acid metabolomics | 1.322 | 2.011 | up |
| 2-(Dimethylamino)Guanosine | Nucleotide metabolomics | 2.559 | 0.456 | down |
| L-Homocystine | Amino Acid metabolomics | 2.629 | 0.287 | down |

**Supplementary Table A4**. Significantly differently expressed metabolites in Met versus control

| Compounds | Class | VIP | Fold Change | Type |
| --- | --- | --- | --- | --- |
| L-Tartaric Acid | Organic Acid And Its Derivatives | 1.508 | 2.921 | up |
| sphinganine | Lipids | 2.953 | 2.740 | up |
| Tryptamine | Tryptamines And Its Derivatives | 2.994 | 2.679 | up |
| N-lactoyl-phenylalanine | Organic Acid And Its Derivatives | 1.179 | 2.634 | up |
| D-Arabitol | Carbohydrate metabolomics | 2.522 | 2.622 | up |
| 3-N-Methyl-L-Histidine | Amino Acid metabolomics | 1.950 | 2.399 | up |
| Lysopc 18:3 | LipidsOthersPhospholipid | 2.818 | 2.315 | up |
| Lysopg 18:1 | LipidsOthersPhospholipid | 2.837 | 2.311 | up |
| Lysopc 18:0 | LipidsOthersPhospholipid | 2.815 | 2.264 | up |
| PAF C-16 | Lipids | 2.809 | 2.259 | up |
| TG(18:0/20:0/22:0) | TG | 2.138 | 2.148 | up |
| Lysopc 17:0 | LipidsOthersPhospholipid | 1.900 | 2.123 | up |
| Lysopc 15:0 | LipidsOthersPhospholipid | 2.084 | 2.123 | up |
| Lysope 18:0 | LipidsOthersPhospholipid | 2.732 | 2.117 | up |
| Lysopc 16:0 | LipidsOthersPhospholipid | 2.564 | 2.037 | up |
| Lysope 16:0 | LipidsOthersPhospholipid | 2.239 | 2.020 | up |
| TRP-GLU | Amino Acid metabolomics | 1.376 | 0.447 | down |
| L-Homocystine | Amino Acid metabolomics | 2.967 | 0.360 | down |

**
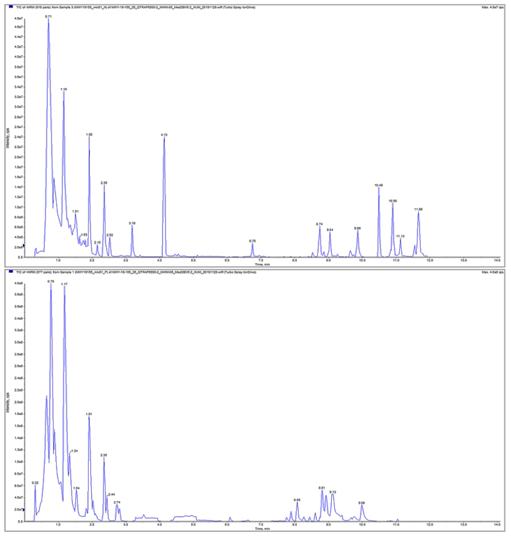

Supplementary Figure A1.** Representative total ion chromatogram (TIC) obtained in electrospray ionization positive ion mode from quality control samples.

**
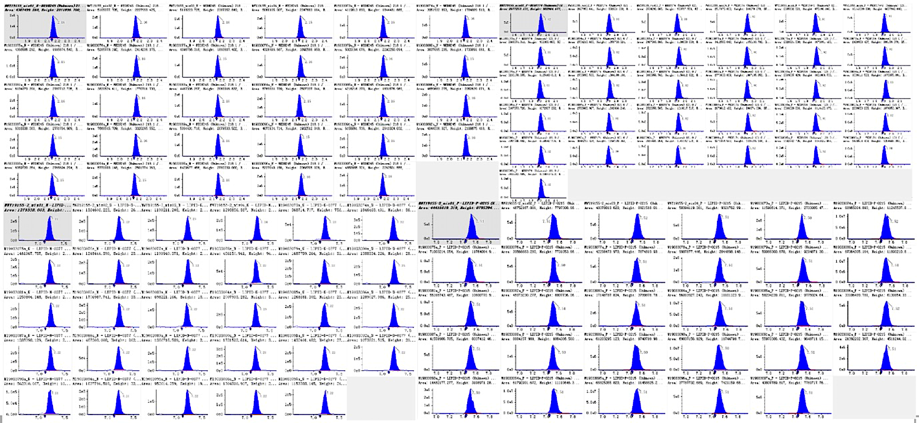

Supplementary Figure A2.** Representative integral correction diagrams for the raw peaks from metabolomics and lipidomics analysis.


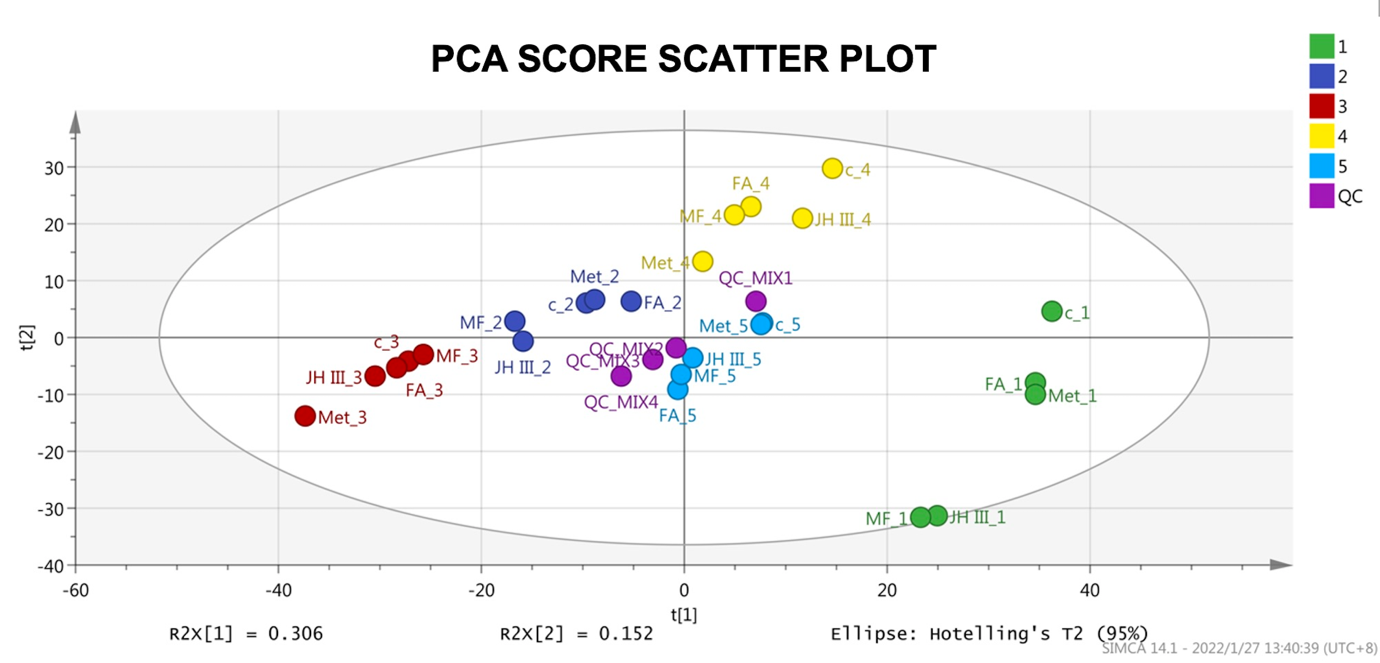
**Supplementary Figure A3.** Score scatter plot for PCA of all groups. *t1 and t2 represent the scores of the first and second PCs respectively, and scatter color represents grouping of the same sample sources, crab 1-5. The score primary ID indicates different treatments. Scores were all within the 95% confidence interval (Hotelling's T2 ellipse).*

**
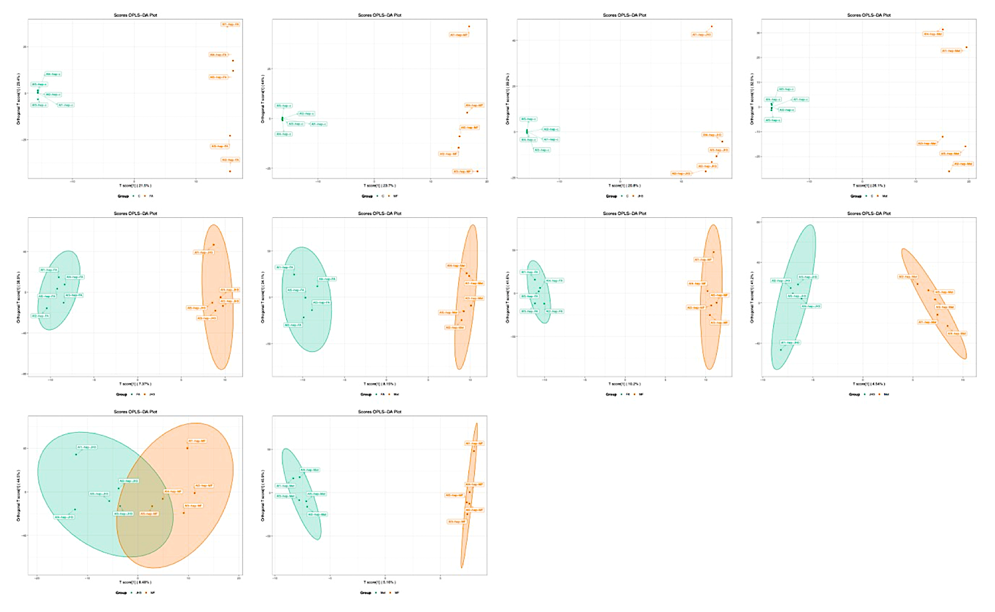

Supplementary Figure A4.** OPLS-DA score scatter plots for comparison groups distinguishing between all treatments.
